# Effects of a Health Promotion Program Based on a Train-the-Trainer Approach on Quality of Life and Mental Health of Long-Term Unemployed Persons

**DOI:** 10.1155/2015/719327

**Published:** 2015-08-25

**Authors:** Heribert Limm, Mechthild Heinmüller, Harald Gündel, Katrin Liel, Karin Seeger, Ramazan Salman, Peter Angerer

**Affiliations:** ^1^Department of Psychosomatic Medicine and Psychotherapy, University Hospital of Ulm, Albert-Einstein-Allee 23, 89081 Ulm, Germany; ^2^Department of Occupational, Social and Environmental Medicine, Ludwig-Maximilians-University, Ziemssenstraße 1, 80336 Munich, Germany; ^3^Ethno-Medical Center e.V. (EMZ), 30169 Hanover, Germany; ^4^Institute for Occupational Medicine and Social Medicine, Centre for Health and Society, Medical Faculty, Heinrich Heine University Düsseldorf, Universitätsstraße 1, 40225 Düsseldorf, Germany

## Abstract

*Background*. Long-term unemployment is associated with poorer mental health. The aim of this study was to evaluate the effectiveness of a health promotion program using the train-the-trainer approach on health-related quality of life (HRQoL) and mental health of long-term unemployed persons. *Methods*. A prospective parallel-group study was conducted among 365 long-term unemployed persons. 287 participants (179 members of the intervention group IG and 108 members of the control group) were reassessed after three months. The intervention comprised both individual sessions based on Motivational Interviewing and participatory group sessions; no health promotion program was administered in the control group. The endpoints were HRQoL (SF-12), depression, and anxiety. The effect size of the change across time in the IG and CG was measured by Cohen's *d*. To assess the significance of group differences in the change across time, a random effects model was used. *Results*. Within three months HRQoL improved and anxiety and depression decreased significantly in the IG. A significant intervention effect was observed for anxiety (*p* = 0.012). Effect sizes in the IG were small to moderate in terms of Cohen's *d* (anxiety: *d* = -0.33; SF-12 mental: *d* = 0.31; depression: *d* = -0.25; SF-12 physical: *d* = 0.19). *Conclusions*. The health promotion program, based on a train-the-trainer approach, showed positive effects on HRQoL and mental health, especially anxiety, of long-term unemployed persons, a highly burdened target group where an improvement in mental health is a crucial prerequisite to social participation and successful reintegration into the job market.

## 1. Introduction

The physical and mental health of the long-term unemployed is considerably worse than that of the working population or the short-term unemployed [1–5]. Thus there is a growing interest in the evaluation of intervention studies to improve health-related quality of life, particularly mental health [1, 2, 6–8], of this highly burdened population group. As in the field of workplace health promotion, the advantage of a setting-based approach, carried out within the welfare organizations in charge of the long-term unemployed, has recently gained increasing attention from experts in this field of research [6, 9].

The longitudinal studies analyzed in a meta-analysis by Paul and Moser in 2009 [2] suggest that unemployment not only is correlated to distress but also leads to deterioration of mental health. Regarding the moderating effect of the duration of unemployment, the results reported in this meta-analysis differ in the level of detail: whereas the cross-sectional data suggests that unemployment duration is a significant negative moderator variable, the longitudinal data shows a curvilinear moderating effect of the duration of unemployment, with renewed worsening of mental health symptoms after 29 months of unemployment. Paul and Moser [2] state that further research efforts are necessary particularly in regard to the very long-term unemployed. Regarding intervention studies, the meta-analysis found that interventions to reduce distress among unemployed persons in general are effective. The effect size, with a value of *d* = −0.35, was medium, showing better mental health outcomes for the intervention groups.

In a randomized controlled trial among 465 long-term unemployed persons in Netherlands, a multidisciplinary intervention which aimed at changing the health complaints of the participants showed no beneficial effects on self-perceived health. The authors suspected that the lack of integration into regular vocational rehabilitation activities (“setting approach”) was the main reason for not seeing any positive effects of the intervention. A process evaluation of the program showed that after the end of the intervention most of the participants resumed their old habits and lifestyle; therefore a continuous “supervision and support” program for this special target group was deemed necessary in order to maintain a healthy and active lifestyle [10].

In summary, there exists some contradictory data on the effects and sustainability of health promotion programs for long-term unemployed persons. The aim of the present study was to evaluate the effectiveness of a setting-based health promotion program on the health-related quality of life and mental health of long-term unemployed subjects in Germany. The intervention was based on a train-the-trainer approach (“supervision and support”) and focused on enhancing the physical activity of the participants as well as their mental health status. The group receiving this intervention was compared to a control group which did not participate in a health promotion program.

## 2. Materials and Methods

A controlled trial with a three-month follow-up was conducted among long-term unemployed persons. Long-term unemployment in Germany is defined as having been unemployed for more than one year and being eligible for benefits according to the SGB-II welfare system. The health promotion program took place in two different settings for the long-term unemployed: in Hanover the program was offered to the long-term unemployed over 50 years of age at a regular job center; in Munich the long-term unemployed were recruited from social organizations participating in the secondary labor market, that is, nonprofit organizations offering employment outside the regular job market. At both settings, participants were assigned either to the intervention or to the control group. In Hanover, assignment to either the intervention group (IG) or the control group (CG) was done by person. In Munich, in each of the nine participating organizations one subunit was independently assigned to the IG, another comparable subunit to the CG.

**Figure 1:**
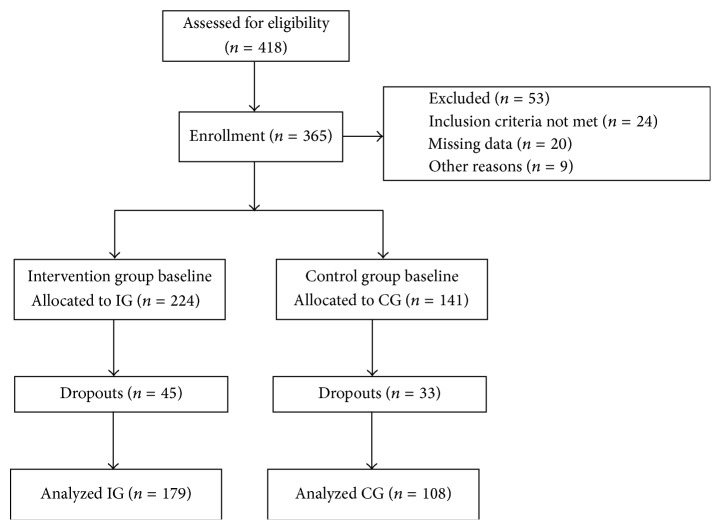
Participant flow during the study and response at 3-month follow-up.

Members of the study team offered information sessions on the health promotion program and the study conditions directly to eligible participants at each setting. A small incentive was given to enhance study participation. Participation in the study was voluntary. Written informed consent was obtained. All volunteers were required to complete a set of questionnaires and participate in a basic medical examination. This health check was conducted by a physician and included feedback to each participant. In total, 418 unemployed persons were eligible at the two study centers and agreed to participate in the study. As 53 persons did not meet the inclusion criteria, at baseline 365 persons (87.3% of those interested) were finally enrolled in the study: 224 in the IG and 141 in the CG (Figure 1). The study was approved by the Ethics Committee of the University of Munich.

### 2.1. Intervention

**Figure 2:**
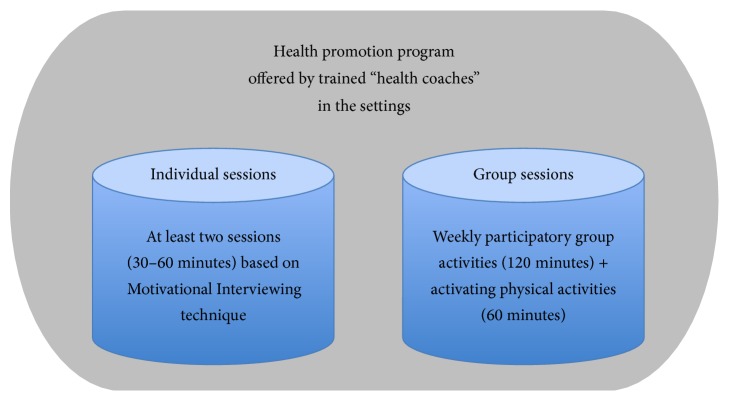
Model of the intervention based on the train-the-trainer approach.

The health promotion program was performed by professionals, mainly social workers or case managers. The program comprised individual sessions based on Motivational Interviewing [11] and participatory group sessions involving physical activity (Figure 2) [12].

The professionals at the two settings were trained by a multiprofessional team in a three-day workshop as “health coaches” to offer the health promotion program at their workplace. To support the implementation of the health promotion program at the local setting, the study team provided continuous supervision for the health coaches. The workshop and the supervision focused on the one hand on evidence-based knowledge of health behavior and on the other hand on skills training, for example, Motivational Interviewing or the instruction of physical activity. The individual sessions of the intervention consisted of at least two meetings within three months dealing with the unemployed person's health behavior. The structure of the individual sessions was based on a previous project which also used Motivational Interviews [13]. The group sessions were planned as weekly participatory activities of two to three hours, dealing with health issues and interests defined by the group participants themselves, for example, organizing a healthy and cheap breakfast at the setting or visiting a fitness trail at the local public park. A detailed description of the health promotion program has been published elsewhere [14].

Members of the control group participated in the health check at baseline. They received feedback on this health check and were asked to complete follow-up questionnaires at the same follow-up time as the intervention group. No health promotion program was administered in the control group.

Both members of the intervention and the control group continued their participation in the usual return-to-work programs offered at their settings during the period of our study.

### 2.2. Outcome Measures

A detailed description of the data collection process has been reported elsewhere [5].

The primary endpoint to assess the effectiveness of the health promotion program was perceived mental (MCS) and physical (PCS) health, measured with the SF-12 questionnaire [15]. The SF-12 is a well-known tool for the assessment of health-related quality of life which has recently been used in several unemployment studies [10, 16, 17]. The Hospital Anxiety and Depression Scale (HADS), which was used to measure the secondary endpoints depression and anxiety, is a validated self-rating instrument for both dimensional and categorical aspects of anxiety and depression [18–20] and has also been used in previous unemployment research [16, 21]. Values between 8 and 10 are judged as signs of clinical anxiety/depression levels and values above 10 as indicators of the need for professional treatment [22].

### 2.3. Determinants of Health and Variables for Subgroup Analyses

The following variables were considered as determinants of health and were set down in advance as relevant variables for subgroup analyses: sociodemographic characteristics (gender, age, duration of unemployment, and migration experience) and study setting. Migration experience was defined as having been born outside Germany. The “setting” variable describes within which setting the participants were recruited, that is, whether the participant was recruited directly at an unemployment center (Hanover) or within the secondary labor market (Munich).

In addition to the variables deemed relevant for subgroup analyses, additional sociodemographic variables and the following health status and health behavior related variables were used for baseline characterization of the study population and for drop-out analyses: body mass index (BMI, kg/m^2^), physical activity, smoking behavior, and alcohol consumption (AUDIT-C score [23]).

### 2.4. Statistical Analyses

As no reference values for PCS and MCS of the SF-12 as primary endpoints in intervention studies with the long-term unemployed were available, a medium effect size of ≤ 0.40 was assumed for the purpose of sample size estimation. For a power of 0.8 and a significance level of 0.05, an estimated sample size of *n* = 100 for each group was calculated. Assuming a drop-out rate of 50%, an overall sample size of 400 was deemed necessary. Participants who completed both the baseline assessment and the three-month follow-up were included in the analysis. The calculation of the PCS and MCS was based on the algorithm provided with the SF-36 manual.

Descriptive analyses were carried out for the analyzed sample at baseline, reporting the mean with SD for numerical data and percentages for categorical data. The reported percentages refer to the number of cases available per variable. Comparisons of the IG and CG were carried out using the Mann-Whitney *U* test for continuous data and Pearson's *χ* ^2^ test for categorical data. The effect size of the change in endpoints across time within the IG and CG was measured by calculating separate Cohen's *d* effect size estimates for both groups based on paired sample *t*-tests. In terms of clinical relevance, an effect size of 0.2 is considered a small effect, 0.5 a moderate effect, and 0.8 a large effect [24].

To assess the significance of group differences in the change across time, a random effects modeling approach with a random intercept term on the subject level and an interaction term for the fixed effects time and group was chosen. The necessity of adjusting for clustering within centers was examined by extending the random effects model to three levels by including a random intercept on the center level. Exploratory subgroup analyses for predefined variables considered as potential confounders and effect modifiers were carried out by including the respective variable and all its interaction terms in the model. For the analyses assessing statistical significance, the predefined significance level was set to 5%. All analyses were carried out in SAS for Windows 9.2.

## 3. Results

Of 365 long-term unemployed persons enrolled in the study at baseline, 287 participants were reassessed three months later, whereas 78 participants completed only the baseline assessments: 45 participants dropped out of the IG and 33 dropped out of the CG. The overall drop-out rate was 21.4% (Figure 1). In the majority of cases (71.4%), the drop-out reason was that the participants had left the setting, including eight persons who had reportedly found employment. In only 13% of all drop-out cases the reason was refusal to participate any longer in the study. In 15.6% of dropouts the SF-12 had not been completed at the three-month follow-up assessment. The remaining 287 participants were included in the three-month evaluation using available cases analysis.

### 3.1. Withdrawal Analysis

Overall, the dropouts and nondropouts showed significant differences only with respect to unemployment duration, migration experience, BMI, and MCS at baseline. In the drop-out group fewer participants reported an unemployment duration of at least five years (38.4% versus 53.6%), but a higher percentage of the dropouts had never worked in Germany (30.1% versus 16.8%). The percentage of participants with migration experience was significantly higher in the drop-out group (51.3% versus 32.1%). The drop-out group also had a significantly lower average BMI (26.5 versus 28.2). The average SF-12 MCS score was significantly higher in the drop-out group (46.9 versus 43.2).

### 3.2. Study Population at Baseline

At baseline in our study sample (*n* = 287) 53.6% had been unemployed for at least five years, and 16.8% had never worked in Germany. 56.1% of participants were women, and the average age of participants was 44.1 years. 21.6% were recruited in the job center setting (Hanover), whereas the great majority (78.4%) of participants were recruited in nonprofit organizations of the secondary labor market (Munich). 32.1% of participants were born outside Germany. A low educational level (respondents left school after less than ten years) was reported by 35.2% of the respondents. 64.2% were not living in a steady relationship with a partner. Our study sample showed low levels of health at baseline in all parameters assessed [5]. A comparison of the IG and CG at baseline revealed significant differences with respect to age, gender, and the SF-12 mental component score (Table 1).

### 3.3. Between-Group Differences and Effect Sizes for SF-12 and HADS

PCS and MCS scores improved significantly in the IG. In the CG both scores also improved, but this increase was not significant. No significant interaction effects were observed at the predefined 5% level. Inclusion of a random intercept term at center level to account for possible confounding by clustering within centres did not change the conclusions. Adjustment for age and gender, the variables for which significant differences between the IG and CG were observed at baseline, had virtually no effect on the results.

In terms of Cohen's *d* effect sizes, for MCS a medium effect size of *d* = 0.31 for the IG and a smaller effect size of *d* = 0.11 for the CG were found at the three-month follow-up. For PCS the effect size after three months was small in both groups, with *d* = 0.19 for the IG and *d* = 0.05 for the CG.

For anxiety a significant time by group interaction effect was observed (*p* = 0.012); that is, the difference between the IG and CG regarding the change over time was significant at the predefined 5% level. The group-specific estimates of the time effect show that this interaction effect was in favor of the IG: In the IG, the anxiety score decreased by 1.03, whereas this score remained virtually unchanged in the CG. For anxiety, the Cohen's *d* effect size was *d* = −0.33 in the IG and *d* = 0.01 in the CG. No significant interaction effect was found for depression; the effect size was *d* = −0.25 in the IG and *d* = −0.06 in the CG (Table 2). As in the case of the outcome variables PCS and MCS, the inclusion of a random intercept term at center level and adjustment for age and gender did not change the conclusions regarding anxiety and depression.

### 3.4. Between-Group Differences for Subgroup Analyses

Subgroup analyses showed some gender effects: in the male subgroup, the MCS improvement in the IG was significantly higher than in the CG (*p* = 0.041 for the time by group interaction effect), whereas, among women, MCS improvement in the IG, though significant as such, was not significantly different from that observed in the CG. Similar gender-specific differences were observed for anxiety, with a significant time by group interaction effect only in the male subgroup (*p* = 0.012). For PCS, on the other hand, a significant time by group interaction effect was observed only among women (*p* = 0.049, with a PCS improvement in the IG as opposed to a decline in the CG). Subgroup analyses by age showed no clear patterns apart from the finding that the only significant time by group interaction effects observed were in the age group 50+ for anxiety and depression (again in favor of the intervention group). With regard to migration experience, significant time by group interaction effects (in favor of the intervention group) were observed for anxiety and depression (*p* = 0.028 and *p* = 0.036, resp.) in the subgroup without migration experience. Subgroup analyses by unemployment duration showed no clear patterns apart from the finding that the only significant time by group interaction effects observed were those for MCS, anxiety, and depression in the subgroup with at least five-year unemployment history (again in favor of the intervention group). Setting was also considered a potential effect modifier beforehand. Subgroup analyses by this variable showed significant time by group interaction effects for MCS and anxiety (in favor of the intervention group) only in the Munich subgroup (Table 3).

### 3.5. Process Evaluation

The process evaluation carried out as an integrated part of the study showed that the intervention was highly accepted by the professionals recruited as health coaches at both settings [14]. In total, 186 Motivational Interviews were performed at baseline and 119 after three months. A total of 209 group intervention sessions were held in the total sample in the first three months, indicating a high degree of compliance with the intervention regime.

## 4. Discussion

This quasi-experimental controlled trial evaluated a novel setting-based intervention to improve health-related quality of life and mental health in a group of long-term unemployed persons in different settings of the German welfare to work system. More than 50% of participants had been unemployed for at least five years.

Three months after the start of the study both the primary and the secondary endpoints had improved significantly in the IG, but not in the CG. For anxiety, the difference between the IG and CG regarding the improvement over time was significant at the 5% level. Subgroup analyses confirmed the positive intervention results with changes in favor of the IG observed especially for men, older participants (50+), and persons with no migration experience and unemployment duration of at least five years. There is also some evidence that participants in a more caring setting (secondary labor market setting in Munich) benefited more from the intervention and that women profited in particular with regard to the physical component score (SF-12).

In this study the within-group effect sizes in the IG were moderate or small whereas in the CG virtually no effects were observed. From a clinical perspective these positive findings in the IG are interesting, as an improvement in the health status of the long-term unemployed is fairly unlikely in the absence of interventions [17, 25] and previous studies have shown that a slight increase in distress scores is a common development among the continuously unemployed [2]. In contrast to our findings, the health promotion program for the long-term unemployed reported by Schuring and his colleagues [8] did not show any beneficial effects on health-related quality of life. Furthermore, a recent study which analyzed the effect of an intensive individual approach in a sample of older long-term unemployed persons only revealed small effects for self-reported health [26]. In comparison with the effect size of *d* = 0.35 for psychological intervention studies among the unemployed reported in the meta-analysis of Paul and Moser [2], the Cohen's *d* effect sizes observed in our study are satisfying. Therefore, the findings presented here indicate that a mix of individual and group interventions can be effective and improve the health-related quality of life and mental health of long-term unemployed persons.

The longitudinal changes observed in the IG of our study sample must be considered a particular success of the health promotion program examined here since, firstly, the very long-term unemployed have often been described as reluctant to change and, secondly, similar medium effects sizes in psychological research are often generated under laboratory conditions and not in welfare settings [27]. Unfortunately, the data reported by Paul and Moser [2] with regard to intervention effects does not provide any specific information on the unemployment duration of the samples analyzed, whereas the sample analyzed in the study presented here consists exclusively of the long-term unemployed. In their meta-analysis, Paul and Moser point out the need for further investigation into the moderator effect of “occupational status.”

The fact that the health promotion program analyzed in our study showed such promising results among the long-term unemployed can, in our view, be explained by the participatory focus in the development of the program (involving both the health coaches at the settings and the unemployed participants) and the continuous supervision and support offered to the health coaches throughout the duration of the program. These aspects of the program design ensured a high degree of empowerment not only at the level of the participants, but also among the professionals in charge of implementing the program in the settings. We believe that these design aspects were major contributors to the high level of acceptance among both the health coaches and the unemployed participants and thereby formed the basis for the success of the health promotion program.

In order to assess the long-term effectiveness of the health promotion program, a 12-month follow-up evaluation of the longitudinal changes in health-related quality of life and mental health was also carried out, the results of which are currently being evaluated.

The promising results of the study can be generalized only with caution. Recruitment of participants was subject to selection into the two settings (job center versus nonprofit organizations of the secondary labor market) and additionally to selection into participation in the health promotion program: the organizations where the intervention was carried out assigned persons (in Hanover) and subunits (in Munich) to the intervention or control group. This organizational aspect was driven primarily by practical reasons; comparability of the respective subunits was ascertained in joint discussions of study team members and staff of the organizations. More subjects were recruited for the intervention group than the control group, which indicates that the recruitment procedures may have favored the enrollment of motivated participants and given rise to instances of self-selection into participation in the health promotion program.

The methodological challenges faced when evaluating social interventions in the field of welfare to work are well documented [28]. Therefore, we attempted to implement a robust, quasi-experimental design. To control for important confounding factors, a comparison group similar in terms of health and unemployment duration was recruited. Unfortunately, in some sociodemographic variables, like age and gender, the matching process failed, with more female and younger participants being recruited in the IG. These differences were controlled for in additional analyses adjusted for age and gender; this adjustment did not lead to a change in the results. However, although both groups were comparable in most key characteristics and outcome parameters, confounding effects due to missing randomization procedures cannot be excluded. Surprisingly, the subgroup analyses showed that more significant intervention effects were attained in older and male subjects, who were underrepresented in the IG. Therefore the overall intervention effects may have been underestimated in this sample. On the other hand, these subgroup results underline the need to further explore the effects of age and gender and to determine whether it is necessary to design age- and gender-specific intervention programs [29].

Another potential limitation is the lack of blinding of the “health coaches,” assessors, and participants in the study. The positive results in favor of the intervention may have been positively influenced by these “nonblinded” conditions.

Finally, the selected endpoints are limited and do not permit an objective assessment of the health status of the participants. However, the use of subjective endpoints as dependent variables was deemed appropriate in view of recent studies [10, 15, 16, 30] which stress the importance of an improvement in self-reported health as an important predictor for successful return to work [31].

The main advantage of our study is the reasonable number of subjects from the group of the long-term unemployed, a population generally judged as noncompliant to both study conditions and health interventions. The setting approach of the intervention enhances the external validity of the results and in combination with the train-the-trainer concept provides the basis for an appropriate tool to install sustainable approaches for health promotion programs in the welfare services.

An aspect of health promotion programs in mental health which is receiving increasing attention is the question of cost-effectiveness. In their assessment of the impact of financial crises on mental health and suggested responses, N. G. Christodoulou and G. N. Christodoulou [32] point out the multifaceted ways and levels in which investing in mental health contributes to cost-effectiveness and increased productivity and encourage mental health professionals to highlight the cost-effectiveness of mental health investments. A limitation of our study in this respect is the fact that the study did not include the systematic collection of quantitative data for assessing cost-effectiveness with standardized instruments. This limitation refers not only to potential beneficial effects in terms of cost-reduction on the one hand (e.g., by reducing the number of health-related days of absence), but also to the implementation costs of the program on the other hand. In particular, an evaluation of the costs incurred by the training sessions for the health coaches and by the intensive supervision and support program would have offered valuable insights into the question of how these costs of the intervention compare to the small to moderate effect sizes observed in terms of improvements in health-related quality of life and mental health.

In the process evaluation of the “train-the-trainer” training program in our study, which was based on Kirkpatrick's four level training evaluation model, we did, however, see various positive indications of the cost-effectiveness of our approach [14]: a case report by one of the participating organizations of the secondary labor market in Munich reported a pronounced decrease in health-related days of absence among their unemployed clients (with a decrease to 4.5% in 2010, the year of the intervention, after levels of 20.4% in 2008 and 18.5% in 2009). Among the professionals trained as health coaches, 84% and 97%, respectively, stated that the techniques of Motivational Interviewing and participatory group sessions were helpful for their daily work. This shows that the health promotion program was well suited to integration in the work processes.

It is widely acknowledged that considerable theoretical and operational deficits remain in the measurement of the (cost-)effectiveness of complex setting-based health promotion programs (see, e.g., [33, 34]). Kirkpatrick [35] points out that the evaluation of training programs with respect to topics such as empowerment is particularly difficult. Future research into the effectiveness of health promotion programs will benefit from finding solutions to these challenges and integrating specific measures of cost-effectiveness (e.g., health-related days of absence) in addition to the evaluation of health outcomes.

## 5. Conclusions

In summary, together with the positive findings reported elsewhere on the effects of this health promotion program in terms of a lifestyle change toward healthier nutrition and more physical activity of the participants [12], the positive changes in the primary and secondary outcome measures of self-reported health after three months provide some evidence of the effectiveness of the intervention. Extended follow-up is required in order to assess whether the expected positive effect of this intervention program on mental health and health-related quality of life actually is sustainable in the long term. There is some evidence that the consideration of subgroups and their needs may enhance the effectiveness of the intervention program. In conclusion, the results of this study indicate that the intervention approach analyzed here may be a promising tool for improving the health status of a highly burdened target group, a group where an improvement in mental health is a crucial prerequisite to social participation and successful reintegration into the job market.

## Figures and Tables

**Table 1:** Subject characteristics: demographic, social, and health behavior variables of the total sample, IG, and CG at baseline^(1)^.

Characteristic	Total	Intervention group	Control group	*p* value^(2)^
	(*n* = 287)	(*n* = 179)	(*n* = 108)	
*Demographic variables *				
Age (years)	44.1 (10.8)	43.1 (10.9)	45.8 (10.4)	0.0336
Gender female	161 (56.1%)	117 (65.4%)	44 (40.7%)	<0.0001
School years				0.4637
<10 years	100 (35.2%)	59 (33.0%)	41 (39.1%)	
10-11 years	84 (29.6%)	57 (31.8%)	27 (25.7%)	
≥12 years	100 (35.2%)	63 (35.2%)	37 (35.2%)	

*Social variables *				
Living in steady relationship	101 (35.8%)	62 (34.8%)	39 (37.5%)	0.6520
Duration of unemployment				0.3504
<5 years	83 (29.6%)	56 (31.6%)	27 (26.2%)	
≥5 years	150 (53.6%)	89 (50.3%)	61 (59.2%)	
Never worked in Germany	47 (16.8%)	32 (18.1%)	15 (14.6%)	
Migration experience	92 (32.1%)	62 (34.6%)	30 (27.8%)	0.2277
Setting job center (versus secondary labour market)	62 (21.6%)	33 (18.4%)	29 (26.9%)	0.0933

*Health-related quality of life (SF-12) and mental health (HADS) variables *				
PCS (SF-12)	44.5 (10.0)	45.0 (9.7)	43.8 (10.3)	0.3846
MCS (SF-12)	43.2 (11.6)	42.0 (11.5)	45.1 (11.4)	0.0273
Depression (HADS summary score)	6.5 (4.3)	6.6 (4.4)	6.3 (4.2)	0.6087
Anxiety (HADS summary score)	7.5 (4.3)	7.7 (4.3)	7.0 (4.2)	0.1833

*Health status and health behavior variables *				
Body mass index (BMI)	28.2 (6.5)	27.9 (6.6)	28.7 (6.2)	0.1486
Physical activity high (at least three times/week)	64 (22.9%)	38 (21.7%)	26 (25.0%)	0.5279
Smoking behavior				0.1342
Smokers	156 (55.5%)	95 (53.7%)	61 (58.7%)	
Never smoked	86 (30.6%)	61 (34.5%)	25 (24.0%)	
Stopped smoking	39 (13.9%)	21 (11.9%)	18 (17.3%)	
Alcohol consumption (AUDIT-C screening test)				0.5853
No consumption (♂: 0; ♀: 0)	97 (34.5%)	65 (36.7%)	32 (30.8%)	
Moderate consumption (♂: 1–4; ♀: 1–3)	121 (43.1%)	73 (41.2%)	48 (46.2%)	
Risk-level consumption (♂: ≥5; ♀: ≥4)	63 (22.4%)	39 (22.0%)	24 (23.1%)	

^(1)^Values are mean (SD) or number of observations (percentage).

^(2)^Mann-Whitney *U* test or Chi^2^ test (categorical data).

**Table 2:** Changes in health-related quality of life (SF-12) and mental health (HADS) after three months.

Variable	Program IG: *n* = 179 CG: *n* = 108	Within-group time effects				Significance of group and time effects (random effects model)		
		Baseline (T1)	After intervention (T2) (+3 months)	Significance of within-group time effect (random effects model)	Effect size	Group effect	Time effect	Group-by-time effect
		Mean (SD)	Mean (SD)	*p* value^(1)^	Cohen's *d* (95% CI)	*p* value^(1)^	*p* value^(1)^	*p* value^(1)^
*Health-related quality of* *life (SF-12) *								
PCS (SF-12)	IG CG	45.0 (9.7) 43.8 (10.3)	46.6 (10.3) 44.2 (9.5)	**0.0092** 0.6133	0.19 (0.04, 0.34) 0.05 (−0.14, 0.24)	0.1035	**0.0453**	0.2265
MCS (SF-12)	IG CG	42.0 (11.5) 45.1 (11.4)	45.3 (11.2) 46.2 (12.1)	**<0.0001** 0.2682	0.31 (0.16, 0.46) 0.11 (−0.08, 0.30)	0.1143	**0.0006**	0.0894

*Mental health (HADS) *								
Depression HADS summary score	IG CG	6.6 (4.4) 6.3 (4.2)	5.8 (4.2) 6.1 (4.4)	**0.0012** 0.5732	−0.25 (−0.40, −0.09) −0.06 (−0.25, 0.13)	0.9337	**0.0144**	0.1169
Anxiety HADS summary score	IG CG	7.7 (4.3) 7.0 (4.2)	6.7 (4.3) 7.1 (4.8)	**0.0001** 0.8688	−0.33 (−0.48, −0.18) 0.01 (−0.18, 0.20)	0.6911	**0.0237**	**0.0118**

^(1)^Unadjusted random effects model; potential effect modifiers were examined in separate subgroup analyses.

**Table 3:** Significance results for subgroup analyses (random effects model).

	Significance of intervention effect (time by group interaction) in different subgroups^(1)^			
	Outcome variable			
	PCS	MCS	Depression	Anxiety
*Subgroup variable *				
Gender				
Female	0.0491	—	—	—
Male	—	0.0406	—	0.0123
Age				
<25 years	—	—	—	—
25–49 years	—	—	—	—
50+ years	—	—	0.0312	0.0194
Migration experience				
Yes	—	—	—	—
No	—	—	0.0361	0.0280
Duration of unemployment				
<5 years	—	—	—	—
≥5 years	—	0.0007	0.0219	0.0003
Never worked in Germany	—	—	—	—
Setting				
Munich (secondary labor market)	—	0.0365	—	0.0204
Hanover (job center)	—	—	—	—

^(1)^ *p* value for significant effects and —: not significant.
